# Tunical plication in the management of penile curvature due La Peyronie’s disease. Our experience on 47 cases

**DOI:** 10.1186/1471-2482-12-S1-S25

**Published:** 2012-11-15

**Authors:** Fabrizio Iacono, Domenico Prezioso, Antonio Ruffo, Ester Illiano, Giuseppe Romeo, Bruno Amato

**Affiliations:** 1 Department of Urology, University “Federico II” of Naples, Via Pansini, 5 - 80131 – Naples, Italy; 2 Department of General, Geriatric, Oncologic Surgery and Advanced Technologies, University “Federico II” of Naples, Via Pansini, 5 - 80131 – Naples, Italy

## Abstract

**Background:**

Peyronie’s disease is an acquired connective tissue disorder of the penile tunica albuginea with fibrosis and inflammation. The disease produces palpable plaques, penile curvature and pain during erections. Patients report negative effects in four major domains: physical appearance and self-image, sexual function and performance. These changes damage sexual life and compromise the quality of life. Our objective is to review the patient's sexual life after penile tunical plicature using the International Index of Erectile Function (IIEF) and the Sexual Encounter Profile (SEP) questionnaires.

**Methods:**

A total of 47 patients with Peyronie's disease (PD) were enrolled at our urology department and they underwent correction of penile deviation between February 2009 and March 2010. Mean patient age was 56 years and mean follow-up was 24 months. Patients with painless PD plaque with no progression in angulation for at least 12 months were chosen for surgery. They underwent a penile tunical plication.

IIEF and SEP questionnaire were administered to all patients.

**Results:**

Of all treated patients, 94% were able to insert their penis in the partner's vagina (p<0.01; SEP question 2), compared with 62% preoperatively and 90% of them was satisfied overall with the sexual intercourse (p<0.01; SEP question 5) .Patients had a significantly higher endpoint and a greater change from baseline for the remaining SEP questions related to achievement of an erection, satisfaction of erection hardness (SEP questions 1 and 4; p < 0:001).

We reported a significant improvement in the IIEF scores (from a baseline total score of 25.2 +/- 3.2 to a final score of 38.3 +/- 5.2; P<0.01). It resulted in significantly higher endpoint IIEF scores across all five IIEF domains: Erectile Function, Intercourse Satisfaction, Orgasmic Function, Sexual Desire and Overall Satisfaction. The main complaint was penile shortening (28 patients, 60%), feeling of the suture during flaccidity and tumescence (37 patients, 80%).

**Conclusion:**

Patient quality of life improved after surgery thanks to the improvement of their sexual life. The complications are unimportant and few bother symptoms are reported. The significant improvement in erectile function was also supported by IIEF and SEP questionnaire data. Nowadays tunical plication is a safe, advantageous and useful technique to treat patients suffering of Peyronie’s disease.

## Background

Peyronie's disease (PD) is a poorly understood connective tissue disorder most commonly attributed to repetitive micro-vascular injury or trauma during intercourse and it is characterized by an inflammatory response beneath the tunica albuginea with fibroblast proliferation where the normal elastic tissue of the tunica is replaced by scar tissue [1] forming a thickened fibrous plaque. The penile plaque, or scar tissue in this condition, is not elastic but hard and will not stretch with erection. The side that does not stretch results in penile curvature on the side of the scar [2].

The prevalence of PD ranges between 3 and 9% of adult men with the most common age of presentation in the fifth decade [3,4]. Recent studies have documented that in 10-15% of men with PD, the condition will resolve spontaneously over 1 year after the diagnosis is made, with 40% remaining the same and approximately 45% progressing [5,6]. Despite progress in the understanding of PD on several fronts, it remains a physically and psychologically devastating condition for the affected patient and partner and it can often lead to an erectile dysfunction [7]. The psychological aspect of both patient and partner are underrepresented in literature; data extracted from select Internet websites have better established PD effects on psyche and relationships [7]. In the presence of severe curvatures, the deformity of the penis interferes with penile penetration, resulting in difficult coitus. Patients with this condition frequently report shame, embarrassment and interpersonal difficulties [8]. Diagnosis is based on medical and sexual histories, which are sufficient to establish the diagnosis. Physical examination includes assessment of palpable nodules and penile length [9]. Curvature is best documented by a self-photograph or pharmacologically induced erection. Prevalence approaches 5%, while less than 20% of men report spontaneous resolution of deformity [7]. Conservative treatment for PD is associated with poor outcomes and surgery is indicated when PD is stable for at least 3-6 months [1]. So far, there have been three basic categories to the surgical approach: shortening procedures on the convex side of the tunica albuginea using tunical excision or plication (Nesbit and its modifications, dot-plication and Yachia procedures); lengthening procedures on the concave side of the tunica albuginea using a graft (with or without plaque incision/excision) are preferred in more severe curvatures or in complex deformities [1]; or implantation of penile prosthesis combined with auxiliary procedures [10]. Essed-Schroeder plication is an established operative technique to correct congenital and acquired penile deviation. However, a third of all patients complain about discomfort from the suture material used [11]. We prospectively evaluated patient satisfaction, quality of life and erectile function after Essed-Schroeder plication by using IIEF and SEP questionnaire.

## Methods

A total of 47 patients with PD were enrolled at our urology department and they underwent correction of penile deviation between February 2009 and March 2010. Mean patient age was 56 years +/- 21 and mean follow-up was 24 months. Patients with painless PD plaque with no progression in angulations for at least 12 months were chosen for surgery. Preoperatively penile curvature was greater than 30 degrees in all patients, as documented by auto-photography using the Kelami technique [12]. Several self-administered patient questionnaires assist in the evaluation of sexual function. One instrument in wide use is the International Index of Erectile Function (IIEF) [13]. A 15-item questionnaire, the IIEF addresses the 5 relevant domains of male sexual function: erectile function, orgasmic function, sexual desire, intercourse satisfaction, and overall satisfaction. The IIEF has been reported to be brief and reliable, psychometrically sound, and easy to administer in both research and clinical settings. It is available (and cross-culturally validated) in 10 languages and demonstrates adequate sensitivity and specificity for detecting treatment-related changes in erectile function [14]. Patients also recorded efficacy information after each sexual encounter by answering 5 yes/no questions in their Sexual Encounter Profile (SEP) diaries. Of primary interest for this study were responses to SEP Question 2: ‘‘Were you able to insert your penis into your partner’s vagina?’’ and SEP Question 3: ‘‘Did your erection last long enough for you to have successful intercourse?’’. Detailed information on possible postoperative discomfort or pain from suture knots, initial irregularities of the penile shaft and penile shortening was provided to the patients preoperatively. Surgery was performed with the patient under epidural anesthesia. The surgical procedure started a coronal incision to 0.5-1cm from the gland line would allow to let intact an adequate amount of reflection of skin (prepuce) bound of preputial skin reflection , maintaining good vascularity and it was completed with a circumcision to prevent foreskin necrosis, phimosis or paraphimosis. A tourniquet was set at the base of the penis. Artificial erection was induced by injection of sterile saline solution into the corpora cavernosa through a 19 gauge butterfly needle to determine the degree of deviation. The careful degloving with preparatory isolation of the dissection plan between dartos and Buck's fascia reduced vascular trauma of the fascia, minimizing bleeding and ensuring tissue vitality. The effect of tunical plication was simulated using Allis clamps with care taken to protect the dorsal neurovascular bundle and its branches. Essed-Schroeder tunical plication using the inverted stitch technique was performed according to Knispel et al. [15]. A 3-zero Polypropylen suture was used for 6 (3 + 3) inverted plication sutures applied contralateral to the maximum point of curvature to straighten the deviation. For ventral curvature the same procedure was performed by subtle isolation and careful lifting of the neurovascular bundles. Artificial erection was then repeated to confirm adequate repair. Buck's fascia is approximated in the midline with interrupted 3-zero quick absorption sutures and skin was closed with interrupted 4-zero quick absorption sutures. Moreover we think that the execution of only two hydraulics erections, after degloving and correction, may cause a minimal tissue stress. At the end a circular dressing with light pressure was applied for 24 hours and a catheter was applied and removed the following day. Patients were advised to have no sexual activity for at least for 6 weeks.

## Results and discussion

In all patients complete anatomical straightening was achieved intra-operatively. Of all treated patients 94% were able to insert the penis in the partner's vagina (p < 0.01; SEP question 2, Table 2) compared with 62% preoperatively. Post-treatment patients also reported a significantly greater proportion of successful intercourse completions (p < 0.01; SEP question 3, Table 2) and 90% of them were satisfied overall with sexual intercourse (p < 0.01; SEP question 5, Table 2). Patients had a significantly higher endpoint and a greater change from baseline for the remaining SEP questions related to achievement of an erection, satisfaction of erection hardness (SEP questions 1 and 4; p < 0:001). We reported a significant improvement in the IIEF scores (from a baseline total score of 25.2 +/- 3.2 to a final score of 38.3 +/- 5.2; P<0.01). It resulted in a significantly higher endpoint of IIEF scores across all five IIEF domains: Erectile Function, Intercourse Satisfaction, Orgasmic Function, Sexual Desire and Overall Satisfaction (Table 1). We noticed only one case of curvature recurrence in a patient who had a sexual intercourse 3 weeks after surgery. 28 patients (60%) reported penile shortening postoperatively, but just 7 of them (15%) complained about that. According to the literature, the most common clinical complaint after plication procedures is penile shortening, even though the majority still have an erectile function. Greenfield et al. [16] pointed out that correction of penile curvatures greater than 60 and/ or a ventral curvature by plication procedures will undoubtedly exacerbate penile shortening after surgery and lead to subjective dissatisfaction. Of all included patients 37 (80%) felt the sutures during flaccidity and tumescence. Discomfort during tumescence occurred in 12 of the 47 patients (25%) whilst 2 patients (4%) had glans hypoesthesia. No patient had wound infection, urethral injury. None of the patients after surgery reported erectile dysfunction or a worsening of their sexual function postoperatively (Table 1 and 2). PD has an important impact over the quality of life of the patients. Patients report negative effects in four major domains: physical appearance and self-image, sexual function and performance, pain and social stigmatization [17]. In 1965 Nesbit introduced corporoplasty with excision of the tunica albuginea and corpus cavernosum from the outer curvature [18]. Pryor and Fitzpatrick popularized this technique for PD. Essed and Schroeder reported tunica plication of the penile convex side 20 years later [19] Many modifications of tunical plication and corporoplasty have been presented [20-22]. The optimal surgical treatment for PD is still debatable [23,24]. The Essed-Schroeder procedure with plication of the tunica albuginea may result in palpable plication nodes with patient discomfort [25]. It can be decreased by using the inverting stitch technique or by varying the suture material [26,27]. In previous studies the definition of subjective postoperative complaints in relation to the suture material used, such as pain, dysesthesia or palpation of plication nodes, was not standardized [24,26]. Results concerning quality of life in regard to the long-term outcome of genital plastic surgery are still scarcely available. To achieve comparable results we used the IIEF and the SEP questionnaire to evaluate the improvement in the sexual life in patients who underwent corporoplasty. This study describes the long-term results of the tunical plications. After more than 24 months of follow-up, we observed that 95% of our patients maintained a straight penis and were satisfied with the procedure. A possible reason for our low rate of recurrence could be plication with 6 sutures. Compared with other studies using non-absorbable sutures our findings show higher satisfaction with suture knots independent of the underlying pathological condition with a lower complication or recurrence rate. Our data provide good results similar to those of previous studies using the inverting stitch technique [25,26]. In 94% of the patients the ability to have sexual intercourse was achieved. Incidentally PD is a risk factor for erectile dysfunction due its organic and psychological affection on male's sexual life. Tunical thickening and intracavernosal fibrosis correlated strongly with difficulty maintaining erection [28] and penile pain represent one of the most distressing, limiting, and difficult to treat manifestations of PD and it can easily lead to erectile dysfunction [29]. Self-reported questionnaires and event-log or daily diary measures are commonly used methods for collecting end-point data in clinical trials of sexual function in men and women [30]. Event-log measures, such as the sexual encounter profile (SEP) [31], offer potential benefits in quantifying the number of intercourse attempts or sexual events and are favored by regulatory reviewers as frequency measures that are potentially less susceptible to recall bias than retrospective self-report questionnaires [32]. SEP has been used as a primary or coprimary end point in registration studies for tadalafil and vardenafil, and a similar diary-based measure was used in earlier registration, so we think that it's a reliable test for evaluating the sexual function after PD surgery. Based on the primary IIEF domain of Erectile Function, mean improvement after surgey was seen by a greater than 17.1 point change in the IIEF total score before surgery, reflecting the importance of penile straightening to improve sexual function in man affected by PD.

**Table 1:** IIEF scores before and after surgery. P value <0.01

Patients N° 47	IIEF Total score	Area 1	Area 2	Area 3	Area 4	Area 5
Before Surgery	21.2 +/ 3.2	4.2 +/- 0.4	5.3 +/- 0.8	4.5 +/- 1.2	4.7 +/- 1.2	2.3 +/- 0.8

After Surgery	38.3 +/- 5.2	10.8 +/- 2.0	5.6 +/- 0.9	6.5 +/- 1.4	9.2 +/- 2.0	6.2 +/- 1.8

Change	17.1 +/- 2.0	6.6 +/- 1.2	0.3 +/- 0.1	2.0 +/- 1.0	4.5 +/- 0.8	3.9 +/- 1.0

Area 1, erectile function; Area 2, orgasmic function; Area 3, sexual desire; Area 4, intercourse satisfaction; Area 5, overall satisfaction

**Table 2:** SEP scores before and after surgery. P value <0.01

Patients (pts) N° 47	N° patients ''yes'' response to SEP 2	N° patients ''yes'' response to SEP 3	N° patients ''yes'' response to SEP 5
Before surgery	28 pts (62%)	20 pts (43%)	12 pts (25%)

After surgery	44 pts (94%)	30 pts (64%)	42 pts (90%)

Change	16 pts (16%)	10 pts ( 21%)	30 pts (75%)

SEP Question 2: ‘‘Were you able to insert your penis into your partner’s vagina?’’

SEP Question 3: ‘‘Did your erection last long enough for you to have successful intercourse?’’

SEP Question 5 : ''Were you satisfied overall with this sexual experience?''

## Conclusions

Patient quality of life was increased after surgery thanks to the improvement of their sexual life. Complications are few and the few bother symptoms regard the penile shortening and suture feeling under penile skin. For the all domains of IIEF, erectile function, intercourse satisfaction, orgasmic function, sexual desire, and overall satisfaction, treated patients had a significantly higher endpoint and a greater change from baseline across all domains compared to results before surgery. The significant improvement in erectile function was also supported by other efficacy measures. Increases in the proportion of positive responses of IIEF and SEP questions concerning patients’ improvement in erections, ability to penetrate their partner, and ability to maintain erection to successful completion of intercourse were significantly higher for treated patients after surgery. Furthermore, based on the SEP questions related to satisfaction of erection hardness and overall satisfaction with the sexual experience in this study, treated patients had a significantly higher endpoint and a greater change from baseline after surgery. So we think that tunical plication is a safe, advantageous and useful technique to treat patients suffering of PD.

## List of abbreviations

PD: Peyronie’s disease; IIEF: International Index of Erectile Function; SEP: Sexual Encounter Profile.

## Competing interests

The authors declare that they have no competing interests.

## Authors’ contributions

FI: conception and design, interpretation of data, given final approval of the version to be published; DP: acquisition of data, drafting the manuscript, given final approval of the version to be published; AR: acquisition of data, drafting the manuscript, given final approval of the version to be published; EI: acquisition of data, drafting the manuscript, given final approval of the version to be published; GR: acquisition of data, drafting the manuscript, given final approval of the version to be published; BA: critical revision, interpretation of data, given final approval of the version to be published
